# Supplementary material for: A Case Study of Chimeric Antigen Receptor T Cell Function: Donor Therapeutic Differences in Activity and Modulation with Verteporfin
Source: Cancers (Basel). 2023 Feb 8;15(4):1085. doi: 10.3390/cancers15041085 (PMC9953964; doi:10.3390/cancers15041085)
Supplement: Supplementary file 1 [file cancers-15-01085-s001.zip › Supplementary Figure S1 and Table S1.pdf]

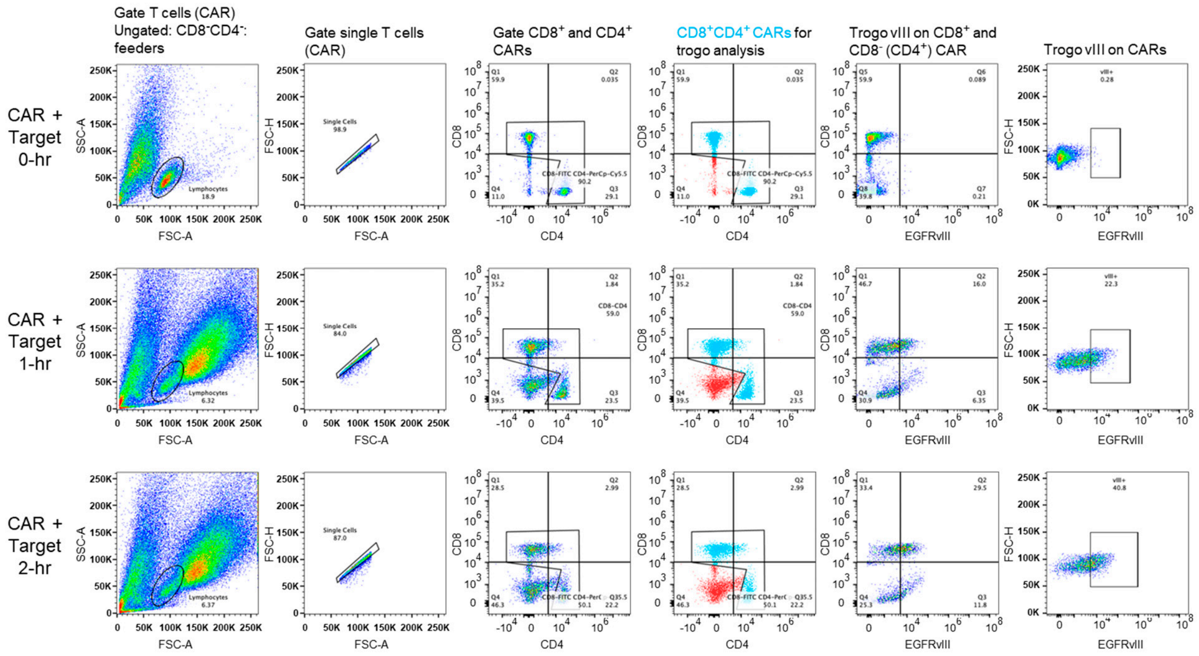

Supplementary Figure S1: **Gating strategy for detecting CAR T cells and acquisition of EGFRvIII (vIII) expression from target co-culture.** Representative flow cytometry analysis of Donor 1. Mixed CAR and target tumors (U87-EGFRvIII) were first defined based on forward (FSC) and side scatter (SSC). A secondary single cell CAR T cell gate was applied. CD4 and CD8 CAR T cell lineages were defined. Thereafter, the expression of EGFRvIII on CAR T cells was quantified.

Supplementary Table S1
Table listing the antibody name, tag, catalog number, and vendor used in all flow cytometry and western blot experiments.

Antibody	Tag	Cat#	Vendor	Application
TIM3	BV421	3545008	BioLegend	Flow
TIM3	Alexa Fluor-488	54669	Cell Signaling Technolgies (CST)	Flow
PD-L1	PE	12-5983-42	ThermoFisher	Flow
PD-L1	APC	17-5983-42	ThermoFisher	Flow
Goat anti-Rabbit	AF647-R-PE	A20991	ThermoFisher	Flow (secondary)
CD14	PE/Cy7	301814	BioLegend	Flow
CD3	Pacific Blue	344824	BioLegend	Flow
CD4	PerCP-Cy5-5	65-0047-T100	TONBO Biosciences	Flow
CD8	BV605	344742	BioLegend	Flow
PD1	APC	329908	BioLegend	Flow
PD1	BV421	329920	BioLegend	Flow
CD57	FITC	555-619	BD Pharmingen	Flow
LAG3	BV650	369316	BioLegend	Flow
EGFRvIII	NA	V3980-100ug	NSJ Bioreagents	Flow/WB (primary)
Goat anti-Rabbit	APC	A10931	ThermoFisher	Flow (secondary)
PD-L1	NA	13684S	CST	WB
Rabbit IgG isotype	NA	3900S	CST	Flow
β-Actin	NA	A1978	Sigma-Aldrich	WB
