# Supplementary material for: A Case Study of Chimeric Antigen Receptor T Cell Function: Donor Therapeutic Differences in Activity and Modulation with Verteporfin
Source: Cancers (Basel). 2023 Feb 8;15(4):1085. doi: 10.3390/cancers15041085 (PMC9953964; doi:10.3390/cancers15041085)
Supplement: Supplementary file 1 [file cancers-15-01085-s001.zip › Supplementary Figure S2.pdf]

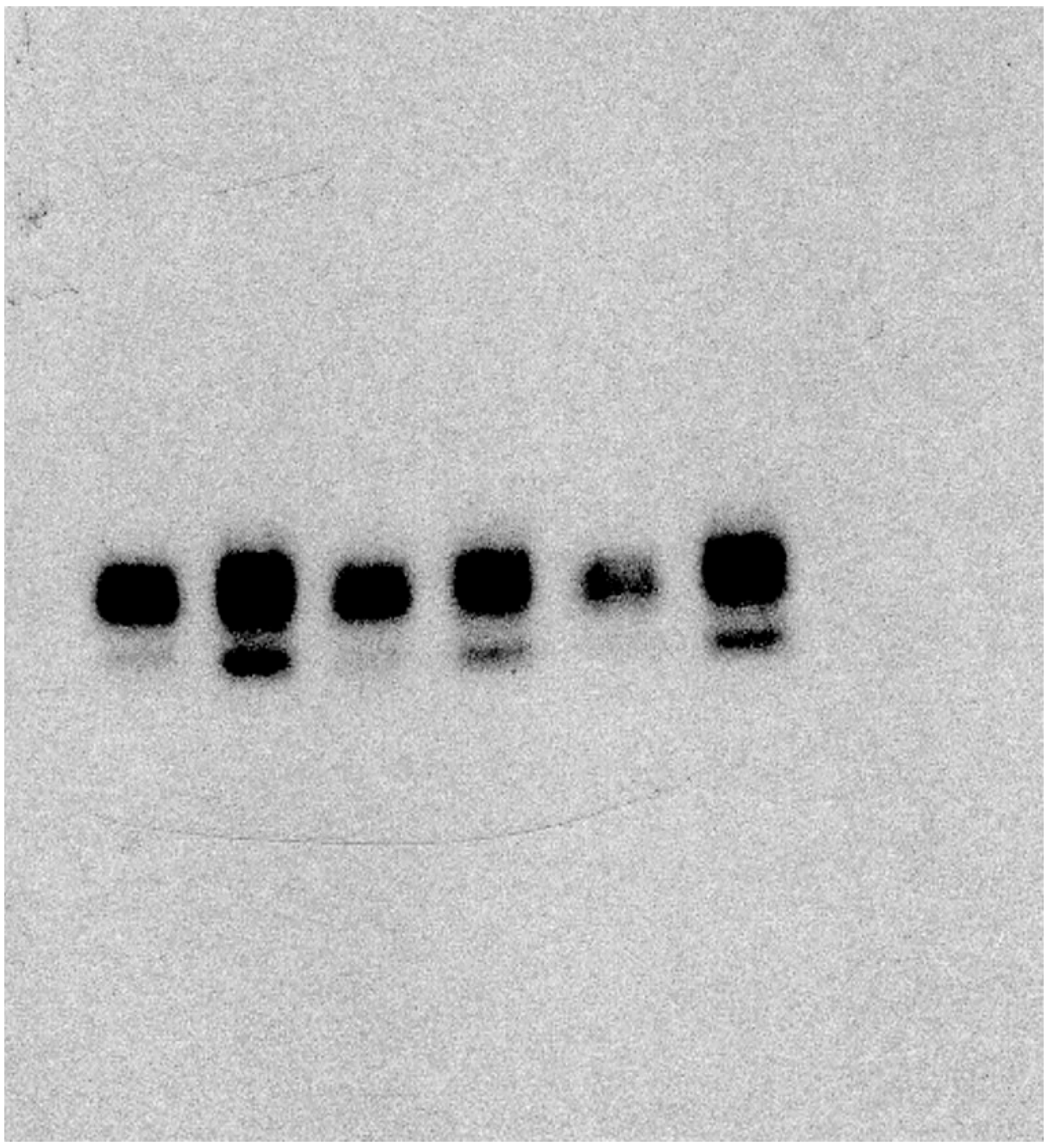

Supplementary Figure S2: **Uncut western blot gel for Figure 6**, showing Baf upregulating PD-L1 on three different donors.
